# Respiratory changes of the inferior vena cava diameter predict fluid responsiveness in spontaneously breathing patients with cardiac arrhythmias

**DOI:** 10.1186/s13613-018-0427-1

**Published:** 2018-08-02

**Authors:** Perrine Bortolotti, Delphine Colling, Vincent Colas, Benoit Voisin, Florent Dewavrin, Julien Poissy, Patrick Girardie, Maeva Kyheng, Fabienne Saulnier, Raphael Favory, Sebastien Preau

**Affiliations:** 1 Intensive care department, Université de Lille, CHU Lille, 59000 Lille, France; 2 Intensive care department, Centre Hospitalier de Valenciennes, 59300 Valenciennes, France; 3 CHU Lille, EA 2694 – Santé Publique : épidémiologie et qualité des soins, Univ. Lille, 59000 Lille, France; 4 Inserm, CHU Lille, U995 – LIRIC – Lille Inflammation Research International Center, Univ. Lille, 59000 Lille, France

**Keywords:** Hemodynamic, Arrhythmia, Atrial fibrillation, Inferior vena cava, Fluid responsiveness, Sepsis, Ultrasound, Echocardiography

## Abstract

**Background:**

Whether the respiratory changes of the inferior vena cava diameter during a deep standardized inspiration can reliably predict fluid responsiveness in spontaneously breathing patients with cardiac arrhythmia is unknown.

**Methods:**

This prospective two-center study included nonventilated arrhythmic patients with infection-induced acute circulatory failure. Hemodynamic status was assessed at baseline and after a volume expansion of 500 mL 4% gelatin. The inferior vena cava diameters were measured with transthoracic echocardiography using the bi-dimensional mode on a subcostal long-axis view. Standardized respiratory cycles consisted of a deep inspiration with concomitant control of buccal pressures and passive exhalation. The collapsibility index of the inferior vena cava was calculated as [(expiratory–inspiratory)/expiratory] diameters.

**Results:**

Among the 55 patients included in the study, 29 (53%) were responders to volume expansion. The areas under the ROC curve for the collapsibility index and inspiratory diameter of the inferior vena cava were both of 0.93 [95% CI 0.86; 1]. A collapsibility index ≥ 39% predicted fluid responsiveness with a sensitivity of 93% and a specificity of 88%. An inspiratory diameter < 11 mm predicted fluid responsiveness with a sensitivity of 83% and a specificity of 88%. A correlation between the inspiratory effort and the inferior vena cava collapsibility was found in responders but was absent in nonresponder patients.

**Conclusions:**

In spontaneously breathing patients with cardiac arrhythmias, the collapsibility index and inspiratory diameter of the inferior vena cava assessed during a deep inspiration may be noninvasive bedside tools to predict fluid responsiveness in acute circulatory failure related to infection. These results, obtained in a small and selected population, need to be confirmed in a larger-scale study before considering any clinical application.

**Electronic supplementary material:**

The online version of this article (10.1186/s13613-018-0427-1) contains supplementary material, which is available to authorized users.

## Background

Acute circulatory failure (ACF) occurs in more than one third of ICU patients [1]. Volume expansion (VE) remains the first treatment provided when ACF of infectious origin is suspected [2, 3]. VE consists of a rapid fluid infusion that aims to increase cardiac output and consequently oxygen transport to tissues. However, cardiac output increase in response to VE, or fluid responsiveness, only occurs when the heart is preload dependent. Increasing evidence of the deleterious effects of inappropriate fluid administration encourages the development of variables predicting fluid responsiveness [4–6], but few have been validated in spontaneously breathing patients, and only exceptionally in patients with irregular cardiac rhythm, frequently excluded from studies [7]. Yet, approximately 25% of septic patients present atrial arrhythmia at ICU admission [8].

The fluid challenge test is the standard reference test although not specifically validated in arrhythmic patients [2]. However, positive response to VE only occurs half a time when the decision is based on clinical criteria, leading to potentially harmful fluid infusion [9]. Thus, the use of tests that do not require fluid infusion remains safer. Because static estimates of preload (e.g., right atrial pressure, pulmonary artery occlusion pressure) do not accurately predict fluid responsiveness, dynamic variables have been developed [7]. Analysis of stroke volume index (SVI) variations or surrogates like arterial pulse pressure variations in response to respiratory-related preload changes is strictly impossible when cardiac rhythm is irregular. Conversely, passive leg raising-induced change in SVI is an accurate variable validated in spontaneously breathing patients with cardiac arrhythmia [10], but may be either technically nonfeasible or unreliable under specific conditions (e.g., pregnancy [11] or intra-abdominal hypertension [12]).

The ability of respiratory-induced variations of the inferior vena cava (IVC) diameters to predict fluid responsiveness has been repeatedly evaluated in spontaneously breathing patients and is still debated because of the potential impact of deep inspiratory movements on the IVC collapsibility [13–15]. In patients without standardization of respiratory efforts, IVC collapsibility (cIVC) predicts fluid responsiveness with high specificity but low sensitivity [13–15]. When assessed during a standardized inspiratory maneuver, cIVC accurately predicts fluid responsiveness in infection-related ACF [15]. However, all patients enrolled in these studies had regular sinus rhythm with no or few chronic cardiac failure or pulmonary hypertension. The arrhythmic cardiac rhythm per se may not interfere with the performances of cIVC to predict fluid responsiveness. Nevertheless, because arrhythmic patients often are older patients with more cardiovascular and respiratory comorbidities, the hemodynamic response to a deep inspiration may change [8, 16, 17]. This study sought to assess the reliability of cIVC to predict preload dependence in the specific population of arrhythmic patients.

We hypothesized that not only cIVC but also the inspiratory diameter of the IVC (iIVC) could predict fluid responsiveness in spontaneously breathing patients with infection-related ACF and irregular cardiac rhythm. We studied the ability of cIVC and iIVC assessed during a standardized inspiratory maneuver to predict fluid responsiveness in these patients. To help the interpretation of the primary outcome, we sought to compare the difference between responders and nonresponders in the IVC diameters variations induced by the variations of the inspiratory effort.

## Methods

### Study design and settings

This prospective two-center study received the regional ethics committee of Nord-Pas-De-Calais, France approval (No. 2011-A00990-41). All subjects received oral and written information and provided written consent prior to study enrollment. All examinations were performed in semirecumbent position with the trunk elevated 30°–45° from the horizontal lower limbs. Ultrasonographic and clinical data were recorded immediately before and after VE, performed as a 30-min infusion of 500 mL 4% gelatin (Gelofusine^®^ 4%, B. Braun, Melsungen, Germany or Plasmion^®^, Fresenius-Kabi, Louviers, France). Relative changes in velocity time integral of aortic blood flow (VTIao) induced by VE were calculated using the formula: VE-induced change in VTIao (%) = 100 × (post-VE value − baseline value)/baseline value. Patients were classified as responders if VTIao increased by ≥ 10%, and nonresponders if VTIao increased by < 10% after VE. This threshold value seemed clinically relevant (i.e., in terms of VE-induced changes in systolic arterial pressure and pulse pressure) and was at least twice the intra-observer variability of the VTIao measured in our previous study [15].

### Selection of participants

Adult patients admitted to the ICUs of the Lille Teaching Hospital and the Valenciennes General Hospital were prospectively screened for inclusion eligibility from May 2012 to May 2015. Patients included in the study were spontaneously breathing patients with irregular cardiac rhythm, hospitalized in ICU for an infection, and for whom a volume expansion has been decided for clinical signs of acute circulatory failure by the physician in charge.

Irregular cardiac rhythm included atrial fibrillation and recurrent atrial extrasystoles > 6/min. Clinical signs of acute circulatory failure were as follows: systemic arterial hypotension (systolic arterial pressure < 90 mmHg, decrease > 40 mmHg from baseline in patients with known hypertension), oliguria (urine output < 0.5 mL/kg/h over 1 h or more), tachycardia (heart rate > 100/min) or mottled skin. In this specific, arrhythmic, spontaneously breathing population hospitalized in our ICUs, preload dependence could only be assessed by a fluid challenge test.

Patients with high-grade aortic insufficiency, impaired transthoracic or abdominal echogenicity, clinical signs of active exhalation, clinical or ultrasonographic evidence of pulmonary edema due to heart failure [18], pregnancy or abdominal compartment syndrome [19] were excluded from the study. The presence of an abdominal compartment syndrome was assessed by clinical examination looking for abdominal pain or specific risk factors proposed by Maluso et al. [19]. When available, an intra-abdominal pressure above 25 mmHg led to the exclusion of the patient.

### Ultrasonographic measurements

Ultrasound examinations were performed using a Vivid-i^®^ or Vivid-S5^®^ (General Electric, Solingen, Germany) echocardiographs in Lille, or a Xario XG^®^ (Toshiba America Medical System INC, Tustin, USA) in Valenciennes, all equipped with a 2–3.5-MHz transthoracic transducer.

Echocardiographic recordings were performed (online) by one of the three expert level (SP, BV and FD) operators [20] and analyzed off-line, on anonymous records, after study completion, at least 3 months after patients inclusion. An advanced level-trained operator [20] (PB, DC, VC), carried out the IVC measurements, blind to clinical and echocardiographic data. One of the two expert level-trained operators who did not perform the initial echocardiography, carried out VTIao and general echocardiographic measurements, blind to clinical data and IVC measurements. Because all the operators were also physicians in the ICU centers, some of them may have remembered some clinical or echocardiographic data. However, all the efforts, including anonymization and tasks partitioning, have been made to keep the operators blind.

After bi-dimensional visualization of the IVC using the subcostal long-axis view, a loop was recorded taking care to maximize the IVC diameter throughout three spontaneous and three standardized respiratory cycles. IVC diameters were measured during spontaneous and standardized respiratory cycles at minimum-inspiratory (iIVC-sp and iIVC-st, respectively) and end-expiratory (eIVC-sp and eIVC-st, respectively) timepoints, within 15–20 mm caudal to the hepatic vein-IVC junction, or 30–40 mm to the IVC–right atrium junction. The cIVC was calculated using the average values from three consecutive respiratory cycles as follows: cIVC (%) = 100 × (eIVC − iIVC)/eIVC under spontaneous (cIVC-sp) and standardized (cIVC-st) breathing. On the same image, the most cephalic point of the liver was identified, and diaphragmatic excursion was estimated as the maximal caudal distance traveled by this point during inspiration [15].

The VTIao was measured by pulsed wave Doppler on a five-chamber apical view [21] during spontaneous respiratory cycles and calculated from the average of 15 consecutive cardiac cycles over one or more respiratory cycles. The aortic valve area was calculated from the average aortic annulus diameter over three cardiac cycles. Both the aortic valve area and the body surface area were considered constant throughout the protocol. SVI was the product of the average value of fifteen consecutive VTIao measurements over one or more respiratory cycles, by the ratio of the aortic valve area to the body surface area.

### Standardized inspiratory maneuver

The standardized inspiratory maneuver was performed as described previously [15]. Briefly, standardized respiratory cycles consisted of a deep standardized inspiration followed by passive exhalation. The deep standardized inspiration consisted of a brief (< 5 s) and continuous inspiration to generate a minimum buccal pressure from − 5 to − 10 mmH2O without any breathing resistor. Buccal cavity pressures were recorded during spontaneous and standardized breathing. Buccal pressure was recorded using a commercially available MP101 micromanometer (0 ± 1000 mmH2O) (KIMO instrument, Montpon, France), connected via a plastic sampling line (usually used as the CO2 sampling line) to an antibacterial filter in series with a S183 mouth end piece (Teleflex Medical, Int’Air medical, Bourg-en-Bresse, France). Of note, only the filter could be responsible for a minimal ventilatory resistance of this system. In addition, diaphragmatic excursion (DiaphExc) was assessed during spontaneous and standardized breathing on ultrasound images using the bi-dimensional mode in subcostal long-axis view as previously described [15]. Briefly, the most cephalic point of the liver, corresponding to end-expiratory diaphragmatic position, was identified, and diaphragmatic excursion was calculated as the maximal caudal distance traveled by this point during inspiration as follows: (horizontal end-expiratory distance between the most cephalic point of the liver and the left screen edge)–(horizontal end-inspiratory distance between the most cephalic point of the liver and the left screen edge).

### Nonechocardiographic hemodynamic variables

The value of all the nonechocardiographic hemodynamic variables (systolic arterial pressure, pulse pressure, heart rate) corresponds to the mean of 3 separate measurements for each patient.

### Outcomes

The primary outcome of the study was to assess the ability of the respiratory-induced variations of the IVC diameters to predict fluid responsiveness in spontaneously breathing patients with cardiac arrhythmia exhibiting ACF related to infection. Two variables were studied to this end: the iIVC-st and cIVC-st, assessed during a deep standardized inspiratory maneuver. To help with the interpretation of the primary outcome, our secondary outcome was to study the difference between responders and nonresponders in the IVC diameter variations (∆cIVC and ∆iIVC) induced by the changes of the inspiratory effort (∆Pinsp and ∆DiaphExc).

### Statistical analysis

Qualitative variables are expressed as count and percentage. Continuous variables are reported as mean ± standard deviation or as median (25th; 75th percentiles) in case of non-Gaussian distribution. Sample size was calculated as previously described [22]. Ninety subjects were needed to predict VE responsiveness with a power of 0.9, a one-sided *Z*-test *p* value of less than 0.05 and an area under the receiver operating characteristic (ROC) curve of iIVC-st greater than 0.7 (the threshold usually considered as acceptable for a diagnostic test) [23]. We anticipated an area under the ROC curve ± standard error of the mean of 0.85 ± 0.15 with a VE responsiveness rate of 40%. Normality of continuous variables was checked graphically and using the Shapiro–Wilk’s test. Data (demographics, clinical characteristics, IVC diameters, respiratory and hemodynamic variables assessed before and after VE) were compared between the two study groups (responders and nonresponders to VE) using the Chi-square test (or Fisher’s exact test when expected cell frequency was < 5) for qualitative variables and the Student’s *t* test (or Mann–Whitney U test for non-Gaussian distribution) for quantitative variables. No statistical comparison was done for qualitative variables with frequency < 5. Comparisons of respiratory variables under spontaneous and standardized ventilation were performed with a paired Wilcoxon test. For each hemodynamic variable, ROC curve analysis was carried out to assess their ability to predict fluid responsiveness. The areas under the ROC curves and their 95% confidence intervals (CIs) were calculated, and optimal threshold values were determined by maximizing the Youden index. Sensitivity and specificity for optimal threshold values were calculated with 95% CIs. In addition, we reported the threshold values to reach a sensitivity or specificity of ≥ 0.90. The absolute VE-induced changes in hemodynamic variables were compared between the two study groups using Student’s t test and analysis of covariance adjusted for baseline values. We assessed correlation between the inspiratory effort and the IVC collapsibility in each responder and nonresponder groups using linear regression analysis. All statistical tests were done at the two-tailed α level of 0.05. Data were analyzed with SAS software version 9.4 (SAS Institute Inc., Cary, NC).

## Results

### Flowchart

Among 85 subjects meeting inclusion criteria, 30 were excluded because of nonmeasurable reference standard velocity–time integral of aortic blood flow (*n* = 17, 20%) or IVC diameter (*n* = 13, 15%). Finally, fifty-five patients were included.

### General variables

Twenty-nine patients had atrial fibrillation (53%) and 26 (47%) frequent extrasystoles. Mean Simplified Acute Physiologic Score II [24] was 36 ± 12. Seven (13%) patients died during hospitalization. The median volume of fluid administered in the 24 h prior to inclusion was 500 (0; 1500) mL. Eight (15%) patients received continuous intravenous norepinephrine at a median dose of 0.34 (0.13; 0.40) μg/Kg/min. Ten (18%) patients had a central venous catheter (i.e., in the superior vena cava area). Intra- and inter-observer variability regarding VTIao was 3.9 ± 2.8% and 8.6 ± 2.5%, respectively. Intra- and inter-observer iIVC-st variability was 0.8 ± 0.8 and 3.6 ± 1.5 mm, respectively. Intra- and inter-observer cIVC-st variability was 8.3 ± 4.1 and 9.8 ± 7.6%, respectively. VE increased VTIao by 16 ± 11% in the whole group, and 29 (53%) patients were responders to VE. Fourteen (54%) and 15 (52%) subjects were responders in the subgroups of patients with atrial fibrillation and frequent extrasystoles, respectively (*p *= 0.88). Baseline patient demographics and treatment-related data are presented in Table 1.

**Table 1:** Baseline characteristics of the patients

	Nonresponders (*n* = 26)	Responders (*n* = 29)	*p* value
*Clinical data*			
Age, year	71 ± 11	66 ± 12	0.12
Sex ratio, female	11 (42)	9 (31)	0.39
Height, cm	168 ± 9	171 ± 10	0.32
Weight, kg	76 ± 23	79 ± 21	0.67
Body mass index, kg/m^2^	27 (23; 29)	26 (22; 29)	0.93
Admission-to-VE time (h)	31 (19; 42)	18 (7; 37)	0.17
*Medical history*			
Chronic systemic hypertension	12 (46)	16 (55)	0.50
Chronic left ventricular failure	5 (19)	9 (31)	0.32
Chronic right ventricular failure	2 (8)	3 (10)	1.00
Chronic obstructive pulmonary disease	6 (23)	8 (28)	0.70
Chronic pulmonary hypertension	5 (19)	3 (10)	0.45
Pulmonary embolism	1 (4)	2 (7)	–
*Infection*			
Pulmonary infections	14 (54)	18 (62)	0.59
Urinary infections	4 (15)	1 (4)	
Abdominal infections	3 (11)	2 (7)	
Skin and soft tissue infections	3 (11)	5 (17)	
Catheter and other infections	2 (8)	3 (10)	
*Treatment*			
Simplified acute physiology score II	39 ± 11	33 ± 13	0.08
Norepinephrine	6 (23)	2 (7)	0.13
VE 24 h before inclusion, mL	500 (0; 2000)	1000 (0; 1500)	0.80
*Clinical hemodynamics variables*			
Atrial fibrillation	14 (54)	15 (52)	0.88
Arterial hypotension	14 (54)	14 (48)	0.68
Tachycardia	18 (69)	23 (79)	0.39
Oliguria	12 (46)	14 (48)	0.88
Mottled skin	4 (15)	8 (28)	0.27

Values are expressed as count (percentage), mean ± standard deviation or median (25th; 75th percentiles)

*VE* volume expansion

### Respiratory variables

The inspiratory maneuver increased the inspiratory depression from − 1 (− 3;− 0.3) mmH2O to − 6 (− 9;− 4) mmH2O (*p *< 0.0001), and the DiaphExc from 8 (6;12) mm to 18 (10;26) mm (*p *< 0.0001), compared to unstandardized spontaneous ventilation. Sixteen (29%) patients could not generate a minimum buccal pressure below − 5 mmH2O, but only five (9%) patients could not generate a minimum buccal pressure below − 3 mmH2O. Respiratory variables are detailed in Additional file 2: Table S1.

### Diagnostic accuracies of IVC collapsibility and diameters

Hemodynamic and echocardiographic variables of the IVC at baseline and after VE in responders and nonresponders to VE are shown in Table 2. The cIVC was significantly greater in responders versus nonresponders (*p *< 0.0001), whereas iIVC and eIVC were significantly smaller (*p *< 0.0001 and *p *< 0.0003, respectively) before VE under both spontaneous and standardized ventilation.

**Table 2:** Hemodynamic variables before and after volume expansion in responders and nonresponders

	Nonresponders (*n* = 26)	Responders (*n* = 29)	*p* value
*VTIao, cm*			
Before VE	15.9 ± 4.5	13.1 ± 3.5	0.01
After VE	16 ± 4.6	17.2 ± 4.9	0.38
*SVI, ml/m* ^2^			
Before VE	29 ± 8	25 ± 8	0.041
After VE	29 ± 8	33 ± 11	0.21
*Systolic arterial pressure, mmHg*			
Before VE	109 ± 22	105 ± 24	0.54
After VE	112 ± 19	118 ± 23	0.23
*Pulse pressure, mmHg*			
Before VE	49 ± 13	44 ± 17	0.26
After VE	50 ± 13	53 ± 19	0.55
*Heart rate, beats/min*			
Before VE	107 ± 30	114 ± 24	0.36
After VE	103 ± 26	107 ± 23	0.97
*cIVC-st, %*			
Before VE	19 (7; 33)	74 (53; 88)	< 0.0001
After VE	11 (8; 20)	36 (22; 61)	< 0.0001
*iIVC-st, mm*			
Before VE	19 (13; 21)	4 (2; 8)	< 0.0001
After VE	21 (19; 23)	13 (8; 16)	< 0.0001
*eIVC-st, mm*			
Before VE	22 ± 4	17 ± 5	0.0003
After VE	24 ± 4	20 ± 5	0.001
*cIVC-sp, %*			
Before VE	11 (8; 32)	49 (25; 66)	< 0.0001
After VE	5 (3; 15)	24 (11; 47)	0.0001
*iIVC-sp, mm*			
Before VE	20 (14; 22)	9 (5; 13)	< 0.0001
After VE	22 (19; 25)	16 (11; 18)	< 0.0001
*eIVC-sp, mm*			
Before VE	23 ± 4	17 ± 5	< 0.0001
After VE	24 ± 4	20 ± 5	0.001

Values are expressed as mean ± standard deviation or median (25th; 75th percentiles). Every single value is the mean of 3 separate measurements

*IVC* inferior vena cava; *cIVC-st* collapsibility index of the IVC under standardized breathing; *iIVC-st* minimum-inspiratory diameter of the IVC under standardized breathing; *eIVC-st* end-expiratory diameter of the IVC under standardized breathing; *cIVC-sp* collapsibility index of the IVC under unstandardized spontaneous breathing; *iIVC-sp* minimum-inspiratory diameter of the IVC under unstandardized spontaneous breathing; *eIVC-sp* end-expiratory diameter of the IVC under unstandardized spontaneous breathing; *SVI* stroke volume index; *VE* volume expansion; *VTIao* velocity time integral of aortic blood flow

**Fig. 1:**
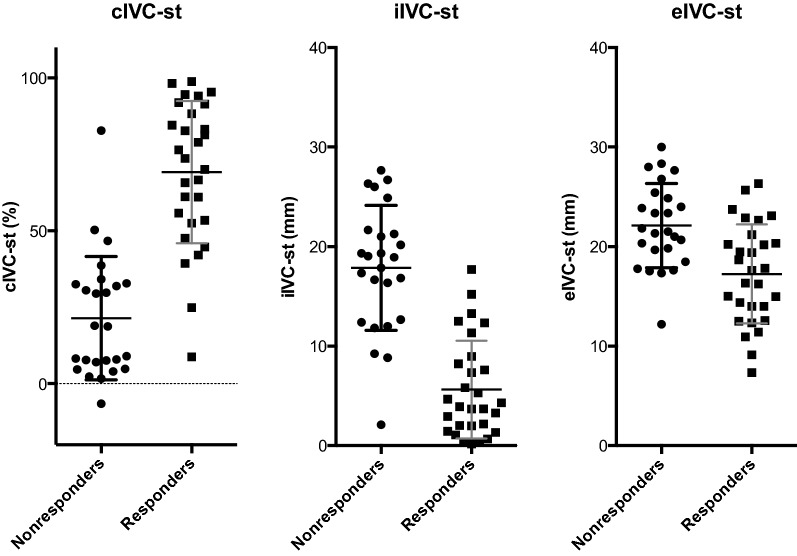
Scatterplot of individual values before volume expansion (VE) for the collapsibility index (cIVC-st), minimum-inspiratory diameter (iIVC-st) and the end-expiratory diameter (eIVC-st) of the inferior vena cava under standardized breathing in responders and nonresponders to VE

The diagnostic performance of cIVC-st, iIVC-st and eIVC-st is presented in Table 3. Individual values of cIVC-st, iIVC-st and eIVC-st before VE are shown in Fig. 1. Area under the ROC curves of cIVC-st and iIVC-st were both 0.93 [95% CI 0.86 − 1] (Additional file 1: Figure S1). A cIVC-st > 39% prior to VE predicted fluid responsiveness with a sensitivity of 0.93 [95% CI 0.77; 0.99] and a specificity of 0.88 [95% CI 0.69; 0.97]. Likewise, an iIVC-st  < 11 mm prior to VE predicted fluid responsiveness with a sensitivity of 0.83 [95% CI 0.64; 0.94] and a specificity of 0.88 [95% CI 0.69; 0.97]. cIVC-st (*R*^2^ = 0.58; *p* < 0.0001) and iIVC-st (*R*^2^ = 0.53; *p* < 0.0001) before VE were correlated with VE-induced change in VTIao (Additional file 1: Figure S2). The performances of cIVC-sp and iIVC-sp before VE to discriminate fluid responders from nonresponders are reported in Table 3, Additional file 1: Figures S1 and S3.

**Table 3:** Accuracy of the inferior vena cava variables for predicting response to volume expansion

Variables	Area under ROC curve [95% CI]	Threshold	Sensitivity [95% CI]	Specificity [95% CI]	Positive predictive value (%)	Negative predictive value (%)
cIVC-st	0.93 [0.86; 1.00]	≥ 39*	0.93 [0.77; 0.99]	0.88 [0.69; 0.97]	89	93
%		≥39	≥0.9		89	93
		≥48		≥0.9	91	84
iIVC-st	0.93 [0.86; 1.00]	< 11*	0.83 [0.64; 0.94]	0.88 [0.69; 0.97]	87	84
mm		<13	≥0.9		76	89
		<9		≥0.9	90	80
eIVC-st	0.77 [0.64; 0.89]	< 19*	0.66 [0.48; 0.83]	0.76 [0.55; 0.91]	73	69
mm		<24	≥0.9		57	82
		<17		≥0.9	92	65
cIVC-sp	0.82 [0.70; 0.93]	≥ 37*	0.66 [0.46; 0.82]	0.85 [0.65; 0.96]	80	70
%		≥11	≥0.9		65	89
		≥43		≥0.9	87	66
iIVC-sp	0.86 [0.76; 0.96]	< 14*	0.79 [0.60; 0.92]	0.81 [0.61; 0.93]	78	82
mm		<20	≥0.9		67	89
		<10		≥0.9	88	69
eIVC-sp	0.83 [0.72; 0.94]	< 18*	0.66 [0.48; 0.83]	0.92 [0.75; 0.99]	90	73
mm		<23	≥0.9		59	83
		<18		≥0.9	90	73

*CI* confidence interval; *IVC* inferior vena cava; *cIVC-st* collapsibility index of the IVC under standardized breathing; *iIVC-st* minimum-inspiratory diameter of the IVC under standardized breathing; *eIVC-st* end-expiratory diameter of the IVC under standardized breathing; *cIVC-sp* collapsibility index of the IVC under unstandardized spontaneous breathing; *iIVC-sp* minimum-inspiratory diameter of the IVC under unstandardized spontaneous breathing; *eIVC-sp* end-expiratory diameter of the IVC under unstandardized spontaneous breathing; *ROC* receiver operating characteristic

*Optimal threshold value to predict response to volume expansion

Because an increase in VTIao with VE ≥ 15% rather than ≥ 10% is frequently used in hemodynamic studies to define responders, the main results of the present study, i.e., baseline characteristics of the patients (Additional file 2: Table S3), hemodynamic variables before and after volume expansion (Additional file 2: Table S4) and the accuracy of the IVC variables to predict fluid responsiveness (Additional file 2: Table S5), are shown in the supplemental material using a VE-related change in VTIao ≥ 15% to define responders.

### VE-induced changes in hemodynamic variables

VE-induced decrease in cIVC-st was greater in responders than in nonresponders (− 26 ± 22% vs. − 8 ± 13%, *p *= 0.001), related to a larger increase in iIVC-st (6 ± 5 vs. 3 ± 3 mm, *p *= 0.013) in unadjusted models, whereas no significant difference was observed for eIVC-st (*p *= 0.**42)** (Additional file 2: Table S2).

### Correlation between inspiratory effort and IVC collapsibility

**Fig. 2:**
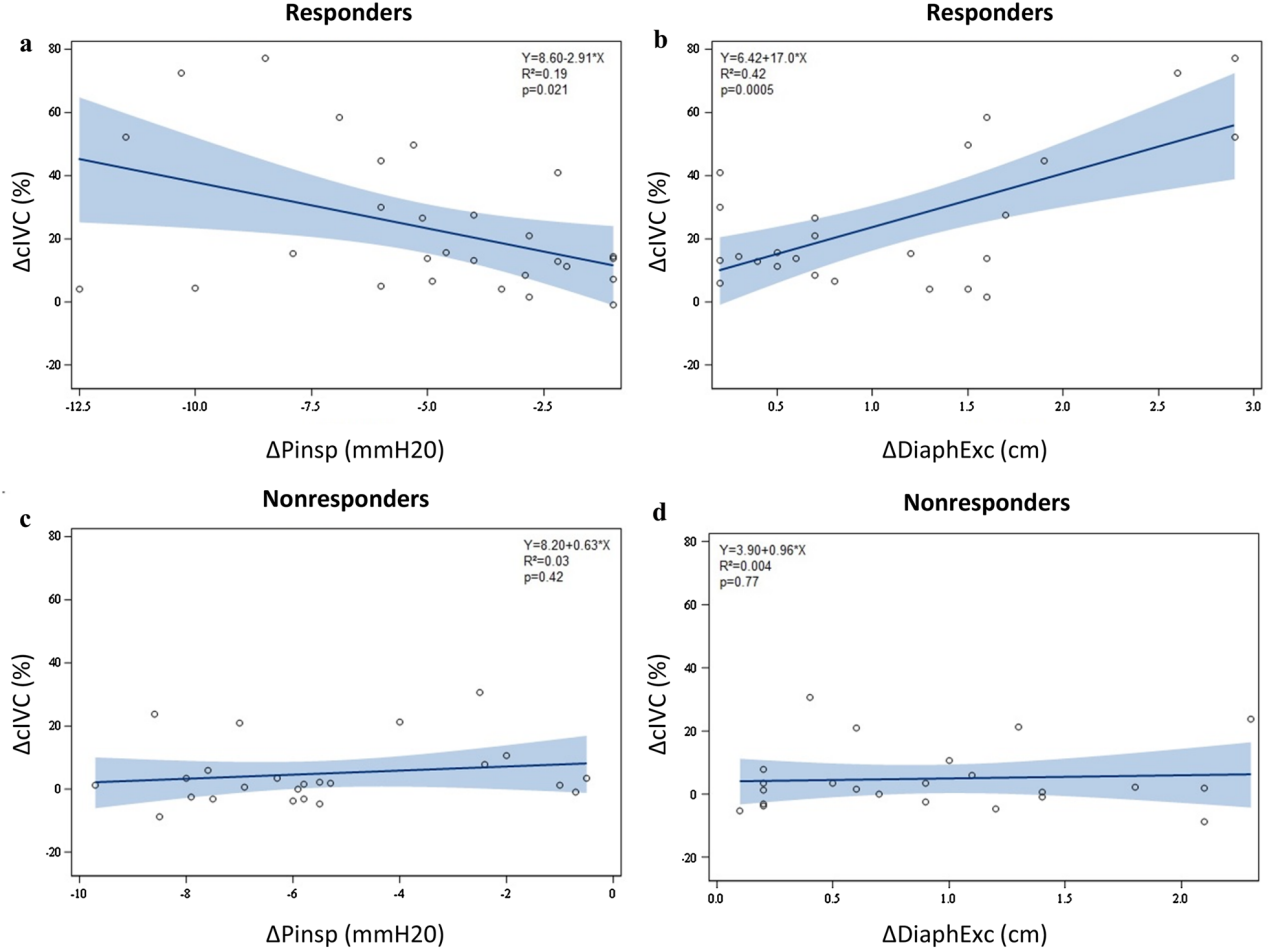
Linear correlation of the delta of collapsibility index of the inferior vena cava (∆cIVC) between standardized and spontaneous unstandardized breathing, and **a** the delta of inspiratory buccal pressure (∆Pinsp), and **b** the delta of diaphragmatic inspiratory excursion (∆DiaphExc) in responders. Linear correlation of ∆cIVC between standardized and spontaneous breathing, and **c** ∆Pinsp, and **d** ∆DiaphExc in nonresponders

**Fig. 3:**
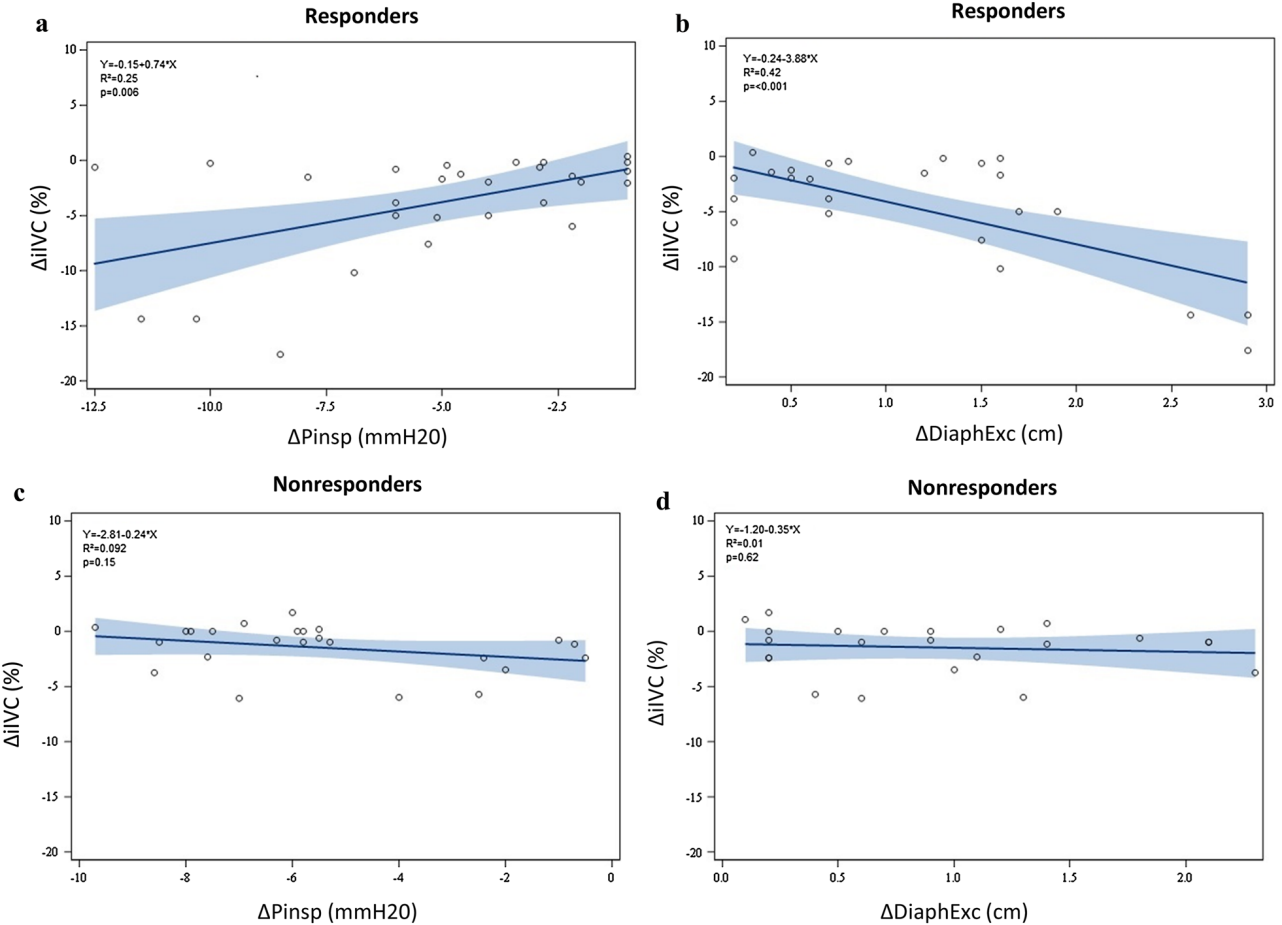
Linear correlation of the delta of the inspiratory diameter of the inferior vena cava (∆iIVC) between standardized and spontaneous unstandardized breathing, and **a** the delta of inspiratory buccal pressure (∆Pinsp), and **b** the delta of diaphragmatic inspiratory excursion (∆DiaphExc) in responders. Linear correlation of ∆iIVC between standardized and spontaneous unstandardized breathing, and **c** ∆Pinsp, and **d** ∆DiaphExc in nonresponders

To assess whether the inspiratory effort similarly impacted cIVC and iIVC in responders and nonresponders, we studied the linear correlations between (1) the standardized inspiration-related changes in cIVC (∆cIVC) or iIVC (∆iIVC) calculated as standardized minus unstandardized values, and (2) the standardized inspiration-related changes in inspiratory pressure (∆Pinsp) or DiaphExc (∆DiaphEx), calculated as standardized minus unstandardized values (Figs. 2 and 3). The ∆cIVC was positively correlated with ∆Pinsp and ∆DiaphEx (*p *= 0.021, *R*^2^ = 0.19 and *p *= 0.0005, *R*^2^ = 0.42, respectively) in responders only, whereas no correlation was observed in nonresponders. The ∆iIVC was negatively correlated with ∆Pinsp and ∆DiaphEx (*p* = 0.006, *R*^2^ = 0.25 and *p* < 0.001, *R*^2^ = 0.42, respectively) in responders, but there was no significant correlation in nonresponders.

## Discussion

To our knowledge, this is the first work reporting the interest of iIVC-st as a potential predictor of response to VE. An iIVC-st < 11 mm predicts fluid responsiveness with a specificity of 88%, a sensitivity of 83% and a negative predictive value of 84%. The gray zone of iIVC-st ranges from 9 to 13 mm. The present study validates that cIVC-st is an accurate predictor of fluid responsiveness in the particular population of spontaneously breathing patients with cardiac arrhythmia during ACF related to infection. A cIVC-st ≥ 39% predicts response to VE with a specificity of 88%, a sensitivity of 93% and a negative predictive value of 93%. The gray zone of cIVC-st is restricted from 39 to 48%. Eventually, both cIVC-st and iIVC-st show good diagnostic accuracy with 95% CI of their area under ROC curve > 0.80.

Studies focusing specifically on fluid management of arrhythmic patients during sepsis are scarce. Because clinical variables cannot predict fluid responsiveness [9] and because fluid overload could be harmful, there is a need for appropriate hemodynamic variables to assess the response to VE in this specific population. In nonventilated, arrhythmic patients, only the fluid challenge [25] and the passive leg raising tests [26] are validated to predict fluid responsiveness. However, the fluid challenge exposes the patients to the risk of inappropriate fluid infusion, and the passive leg raising cannot be performed with all beds and stretchers, raising the issue of the feasibility in clinical routine [3, 13]. The search for predictive factors of fluid responsiveness in our study population was relevant because VE decided on clinical variables increased VTIao in 53% of patients only. In addition, the very close rate of responders in patients with atrial fibrillation (54%) and frequent extrasystoles (52%) justified to gather these two populations of infected patients in the analysis. With regard to their often older age and greater comorbidities including cardiac insufficiency [8, 16, 17], arrhythmic patients may present an increased risk of deleterious effects induced by inappropriate VE [27–30]. In case of acute circulatory failure related to an infection, VE is frequently performed even though dynamic tests do not clearly support the decision [3]. Thus, clinicians need tools with high negative predictive value to avoid potentially harmful VE, rather than yet another incentive to initiate VE.

With regard to unstandardized spontaneous ventilation, our data show a high specificity of cIVC-sp but a low sensitivity to predict response to VE, as previously published in infected patients with regular sinus rhythm [13–15]. Indeed, in patients with regular cardiac rhythm, Muller et al. [13], Airapetian et al. [14] and our team [15] reported that cIVC-sp > 40%, > 42% and > 31% were predictive of fluid responsiveness with high specificity of 80, 97 and 88% but low sensitivity of 70, 31 and 76%, respectively. The similarities in the results from these studies despite different study populations and measurement techniques support the reliability of IVC variations to predict fluid responsiveness with high specificity in septic patients, regardless of their cardiac rhythm. Although the contribution of cIVC-st is small in case of high cIVC-sp values, performing an inspiratory maneuver seems of interest when cIVC-sp values are low. Indeed, in our selected population, using a deep inspiratory maneuver when assessing cIVC to predict fluid responsiveness allows the reduction of false-negative responses (10 in spontaneous unstandardized breathing vs. 2 in standardized inspiration) without creating false positive (4 in spontaneous unstandardized breathing vs. 3 in standardized inspiration). These results suggest that a deep inspiration might significantly improve cIVC sensitivity and negative predictive value to detect fluid responsiveness, without altering specificity in arrhythmic patients. These results are consistent with those of our previous work on cIVC-st in infected critically ills with regular cardiac rhythm [15]. In this population, a cIVC-st ≥ 48% predicted response to VE with a specificity of 90% and a sensibility of 84%, and a gray zone ranging from 39 to 48%. Like cIVC, a deep standardized inspiration maneuver improves fluid responsiveness prediction of iIVC with a 95% CI of its area under ROC curve increasing from < 0.80 to > 0.85.

One limitation frequently discussed about the use of the respiratory changes of the IVC diameter to predict fluid responsiveness in spontaneously breathing patients is the impact of an uncontrolled inspiratory effort on the vessel collapsibility, questioning the reliability of this variable [31]. Based on a physiological study performed on healthy volunteers (i.e., responders to VE [32, 33]), it has been shown that the IVC collapsibility was affected by the inspiratory effort [31] with potential risk of false-negative or false-positive responses when the inspiratory effort is, respectively, insufficient or excessive. In our study, the decrease in false-negative responses with the use of the deep inspiratory maneuver suggests that an insufficient inspiratory effort might actually be responsible for a lack of sensitivity. However, the fact that no false-positive response occurred with the use of the inspiratory maneuver, together with the absence of correlation between the IVC collapsibility and the intensity of the inspiratory effort in nonresponders, may suggest that a deep inspiration might be unlikely to increase the collapsibility of the IVC in nonresponders, contrarily to responders. These specific findings need to be confirmed in larger studies. Similarly, it has been shown in the literature that the IVC collapsibility was dependent on the sampling zone. The IVC percentage collapse at the junction of the right atrium and IVC was dissimilar to the other sites of measurement (hepatic or renal). Thus, it is recommended not to use this proximal sampling zone to assess IVC diameters respiratory variations. Subsequently, all the measurements in our study were performed within 15–20-mm caudal to the hepatic vein–IVC junction, or 30–40 mm to the IVC–right atrium junction [34].

As previously described, 16 (29%) patients were unable to reach the predefined inspiratory pressure threshold of − 5 mmHg [15]. A smaller inspiratory target (e.g., − 3 mmHg) may be proposed for clinical use, as only 5 (9%) patients were unable to reach this threshold value. Interestingly, 2 of the 3 patients unable to reach an inspiratory pressure below − 3 mmHg were classified as false negative with the cIVC-st test. Similarly, 3 patients over the 6 classified as false negative with the iIVC-st test did not reach the − 3 mmH2O threshold. Thus, negative results of cIVC-st and iIVC-st should be carefully interpreted in patients unable to perform an adequate inspiratory effort. These results highlight the importance of an adequate inspiratory maneuver, meaning a deep (< − 3mmH20), brief (< 5 s), continuous and regular inspiratory strain to enhance the diagnostic performance of cIVC and iIVC.

Interestingly, although not statistically significant, the nonresponders show a trend toward older age and higher severity with regard to SAPS2 values and norepinephrine infusion, compared to the responders. However, these criteria were not discriminant enough to help in the prediction of fluid responsiveness. The reasons for this trend remain unknown and cannot be explained by any data collected in this work.

This study has several limitations. First, fifty-five patients were included, instead of the 90 anticipated in sample size calculation. This could be at least partly explained by a lower frequency of patients meeting the inclusion criteria than expected, and the need for an available operator on site to perform the inclusion and the initial echocardiography. Nevertheless, the areas under ROC curve of cIVC-st and iIVC-st were greater than those anticipated in the sample size calculation. Thus, the final sample size had enough power to demonstrate that cIVC-st and iIVC-st before VE have a good diagnostic accuracy to predict fluid responsiveness with 95% CI of their areas under ROC curve > 0.80. However, the representativity of the population could be questioned as only a small number of patients has been included and does not allow any generalization of the conclusions to other populations. For these reasons, this study may rather be considered as a pilot study, especially for the iIVC variable that has never been studied before. Second, the assessment of our dynamic variables has been performed in a very selected population, as only patients with infection-related ACF, with no or low-dose of norepinephrine, for whom VE has already been decided by the physician in charge, were enrolled. Plus, patients with perturbations of intra-abdominal pressure observed in active exhalation, abdominal compartment syndrome, pregnancy and other specific conditions that alter sonographic images like obesity or abdominal surgery which could have interfered with cIVC accuracy were excluded from the study. Therefore, our results cannot be generalized to an unselected critically ill population. Concerning patients’ inclusion or exclusion criteria, tachycardia might be poorly appropriate to detect ACF in arrhythmic patients, and other nonclinical markers, like lactate, pCO_2_ gap or central venous oxygenation, could have been helpful to refine the screening of the patients, although requiring adequate arterial and central venous catheter which are not always available. Likewise, assessing the intra-abdominal pressure along with clinical examination might have been more appropriate to detect intra-abdominal hypertension. Third, IVC diameters were not measurable in 15% of the patients because of a lack of echogenicity, raising the question of the practical application of these variables to all patients. Fourth, although all the efforts have been made to maintain the operators blind, some of the operators may have remembered some clinical or echocardiographic data. Fifth, we arbitrarily defined the positive response to VE as an increase in VTIao of ≥ 10% with rapid fluid loading. This threshold value seems clinically relevant and is more than twice as high as the value of the intra-observer variability of the VTIao measured in this study. Eventually, for feasibility reasons, we did not assess intra-abdominal and central venous pressures, which would have been highly helpful to understand the underlying physiological mechanisms involved in the respiratory variations of the IVC. Last, although IVC diameter changes throughout the cardiac cycle [35], IVC measurements were not taken with electrocardiogram synchronization to detect tele–diastole as usually recommended. This uncertainty in the end-diastolic measurement of the expiratory diameter of the IVC possibly impairs the diagnostic accuracy of cIVC but improves clinical feasibility.

## Conclusions

In a small and selected population of spontaneously breathing patients with cardiac arrhythmia, the collapsibility index and the inspiratory diameter of the IVC accurately predict fluid responsiveness during infection-related acute circulatory failure. A standardized, deep inspiration might improve their sensitivity to detect fluid responsiveness without altering their specificity. The simplicity and rapidity of the measurement of the inspiratory diameter of the IVC may make it a useful tool for fluid management at patients’ bedside. However, the several limitations raised in this work should lead to a cautious interpretation of the results, which need to be confirmed in a larger-scale study before considering any clinical application.

## Additional files

**Additional file 1: Figure S1.** **A**, Receiver operating characteristics (ROC) curve of the collapsibility index (cIVC-st) and the inspiratory diameter (iIVC-st) of the inferior vena cava during a standardized inspiratory maneuver before volume expansion (VE) to discriminate responders from nonresponders to VE in the overall population. **B**, ROC curve of the collapsibility index (cIVC-sp) and the inspiratory diameter (iIVC-sp) of the inferior vena cava during unstandardized spontaneous breathing before VE to discriminate responders from nonresponders to VE in the overall population. **Figure S2. A**, Linear correlation between the collapsibility index of the inferior vena cava under standardized breathing (cIVC-st) before volume expansion (VE) and VE-induced change in the velocity time integral of aortic blood flow (VTIao). **B**, Linear correlation between the inspiratory diameter of the inferior vena cava under standardized breathing (iIVC-st) before VE and VE-induced change in VTIao. **Figure S3.** Scatterplot of individual values before volume expansion (VE) for the collapsibility index (cIVC-sp), minimum-inspiratory diameter (iIVC-sp), and the end-expiratory diameter of the inferior vena cava (eIVC-sp) under unstandardized spontaneous breathing in responders and nonresponders to VE.

**Additional file 2: Table S1.** Respiratory variables in responders and nonresponders before and after volume expansion. **Table S2.** Volume expansion-induced changes in hemodynamic variables in responders and nonresponders. **Table S3.** Baseline characteristics of the patients (VE-related change in VTIao ≥ 15% to define responders). **Table S4.** Hemodynamic variables before and after volume expansion in responders and nonresponders (VE-related change in VTIao ≥ 15% to define responders). **Table S5.** Accuracy of the inferior vena cava variables for predicting response to volume expansion (VE-related change in VTIao ≥ 15% to define responders).

## Abbreviations

ACF: acute circulatory failure
CI: confidence interval
cIVC: collapsibility index of the inferior vena cava
CVP: central venous pressure
*∆*: values recorded during standardized breathing minus values recorded during unstandardized spontaneous breathing
DiaphExc: diaphragmatic inspiratory excursion
eIVC: expiratory diameter of the inferior vena cava
ICU: intensive care unit
iIVC: inspiratory diameter of the inferior vena cava
IVC: inferior vena cava
Pexp: expiratory buccal pressure
Pinsp: inspiratory buccal pressure
ROC: receiver operating characteristic
sp: spontaneous unstandardized breathing
st: standardized breathing
SVI: stroke volume index
VE: volume expansion
VTIao: velocity time integral of aortic blood flow
