# Supplementary material for: Respiratory changes of the inferior vena cava diameter predict fluid responsiveness in spontaneously breathing patients with cardiac arrhythmias
Source: Ann Intensive Care. 2018 Aug 2;8:79. doi: 10.1186/s13613-018-0427-1 (PMC6072642; doi:10.1186/s13613-018-0427-1)
Supplement: Supplementary file 1 — Additional file 1: Figure S1. A, Receiver operating characteristics (ROC) curve of the collapsibility index (cIVC-st) and the inspiratory diameter (iIVC-st) of the inferior vena cava during a standardized inspiratory maneuver before volume expansion (VE) to discriminate responders from nonresponders to VE in the overall population. B, ROC curve of the collapsibility index (cIVC-sp) and the inspiratory diameter (iIVC-sp) of the inferior vena cava during unstandardized spontaneous breathing before VE to discriminate responders from nonresponders to VE in the overall population. Figure S2. A, Linear correlation between the collapsibility index of the inferior vena cava under standardized breathing (cIVC-st) before volume expansion (VE) and VE-induced change in the velocity time integral of aortic blood flow (VTIao). B, Linear correlation between the inspiratory diameter of the inferior vena cava under standardized breathing (iIVC-st) before VE and VE-induced change in VTIao. Figure S3. Scatterplot of individual values before volume expansion (VE) for the collapsibility index (cIVC-sp), minimum-inspiratory diameter (iIVC-sp), and the end-expiratory diameter of the inferior vena cava (eIVC-sp) under unstandardized spontaneous breathing in responders and nonresponders to VE. [file 13613_2018_427_MOESM1_ESM.pptx]

## Slide 1
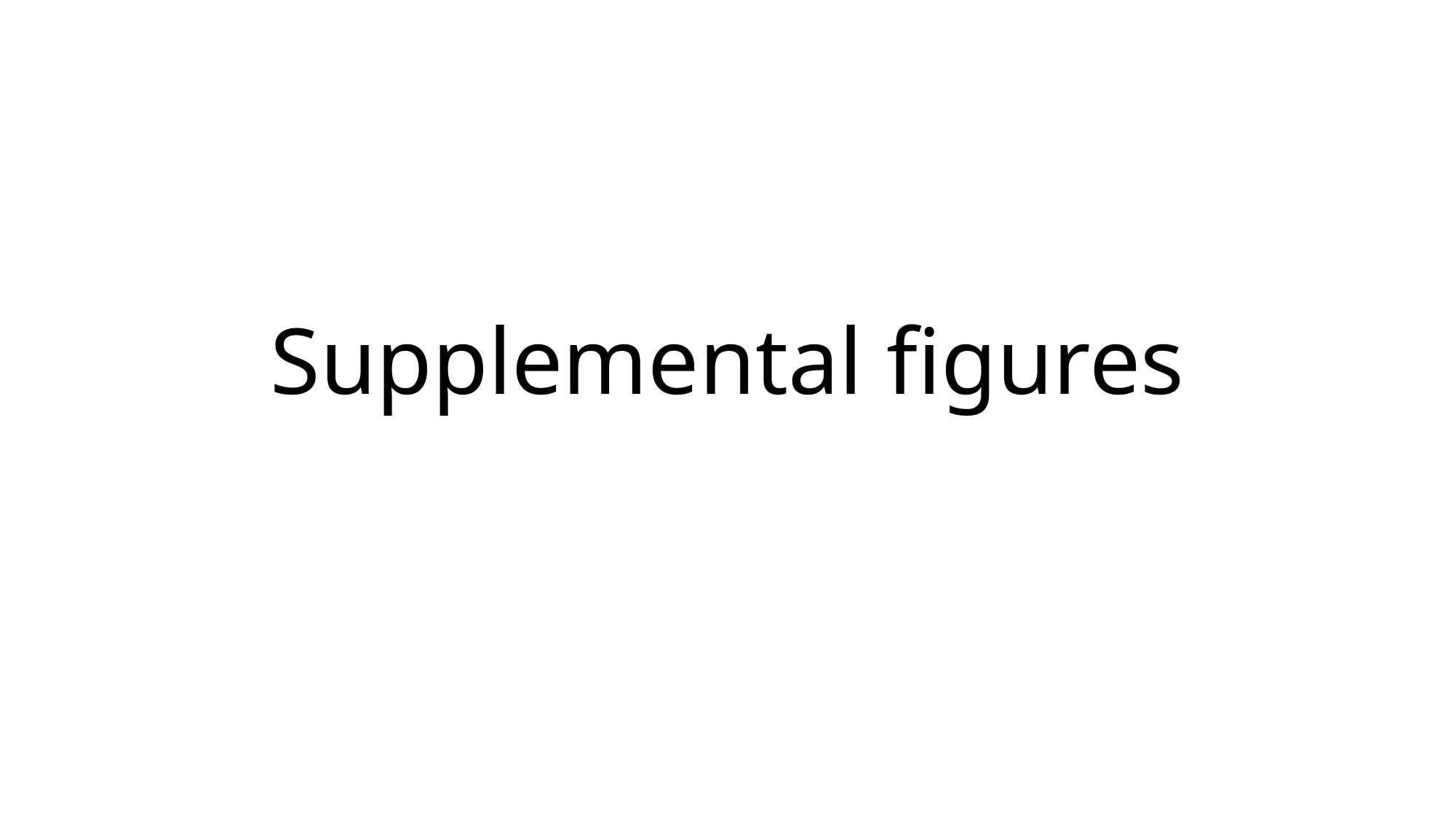

# Supplemental figures

## Slide 2
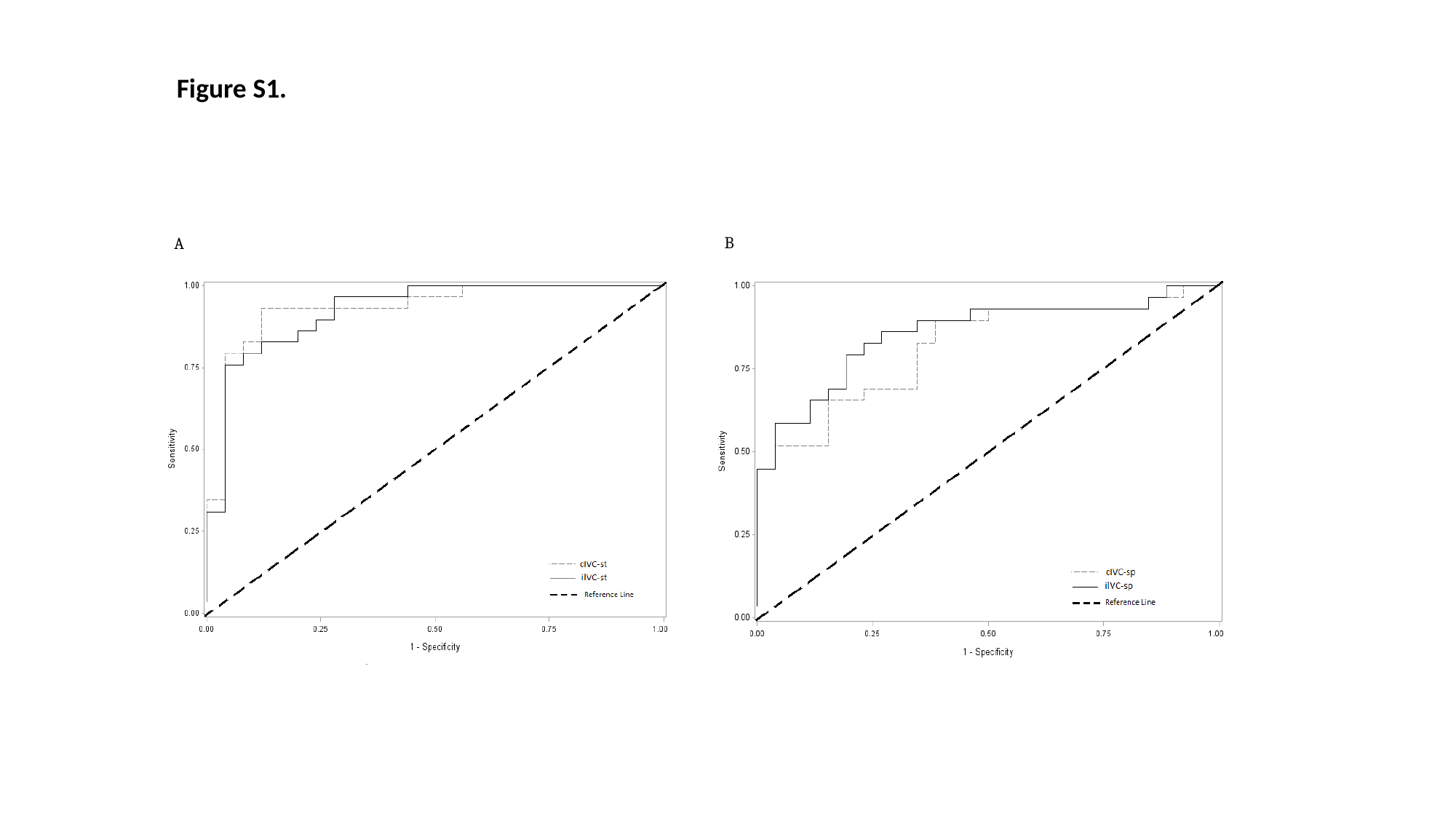

Figure S1.
B
A

## Slide 3
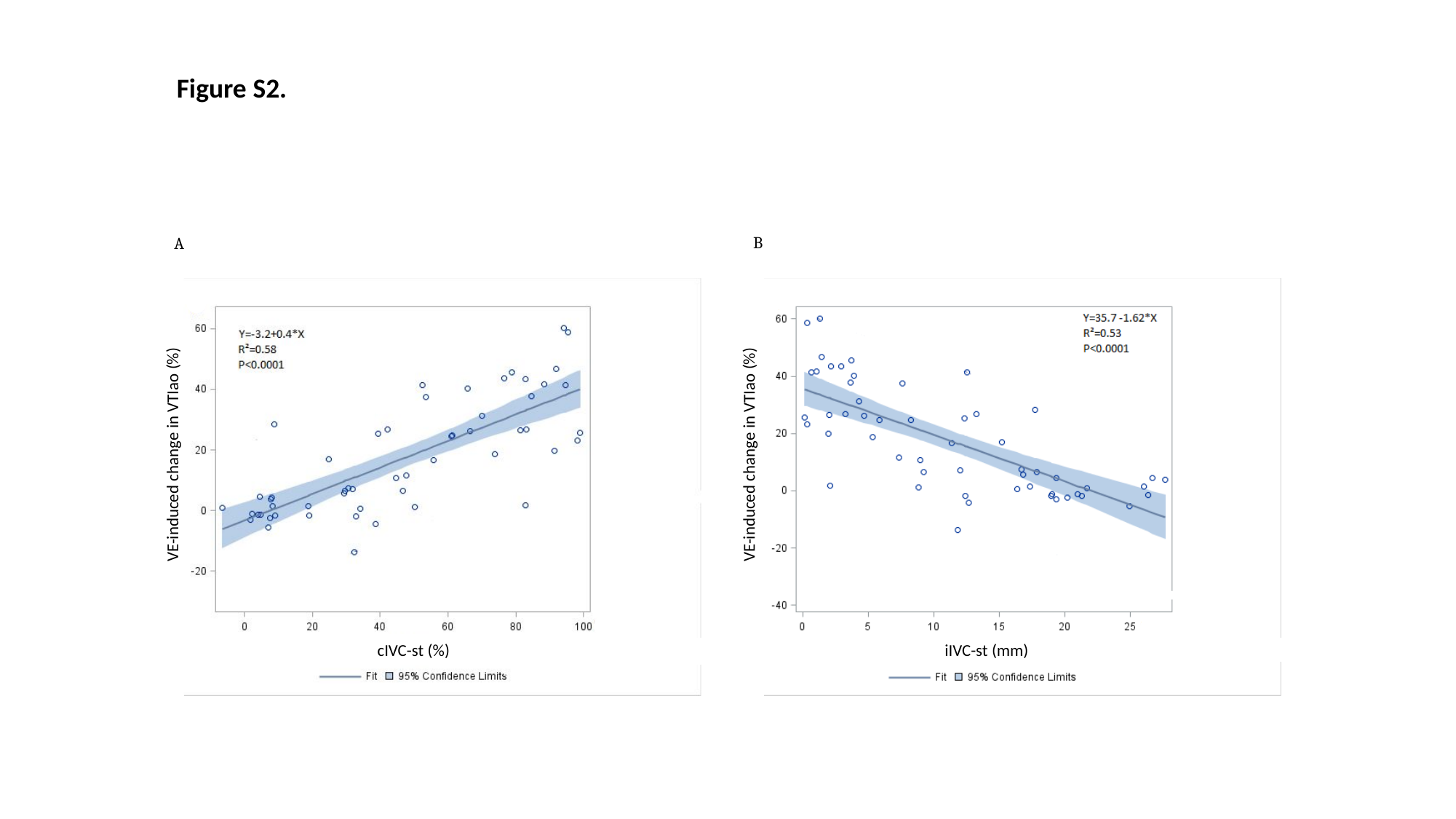

Figure S2.
B
A
VE-induced change in VTIao (%)
VE-induced change in VTIao (%)
cIVC-st (%)
iIVC-st (mm)

## Slide 4
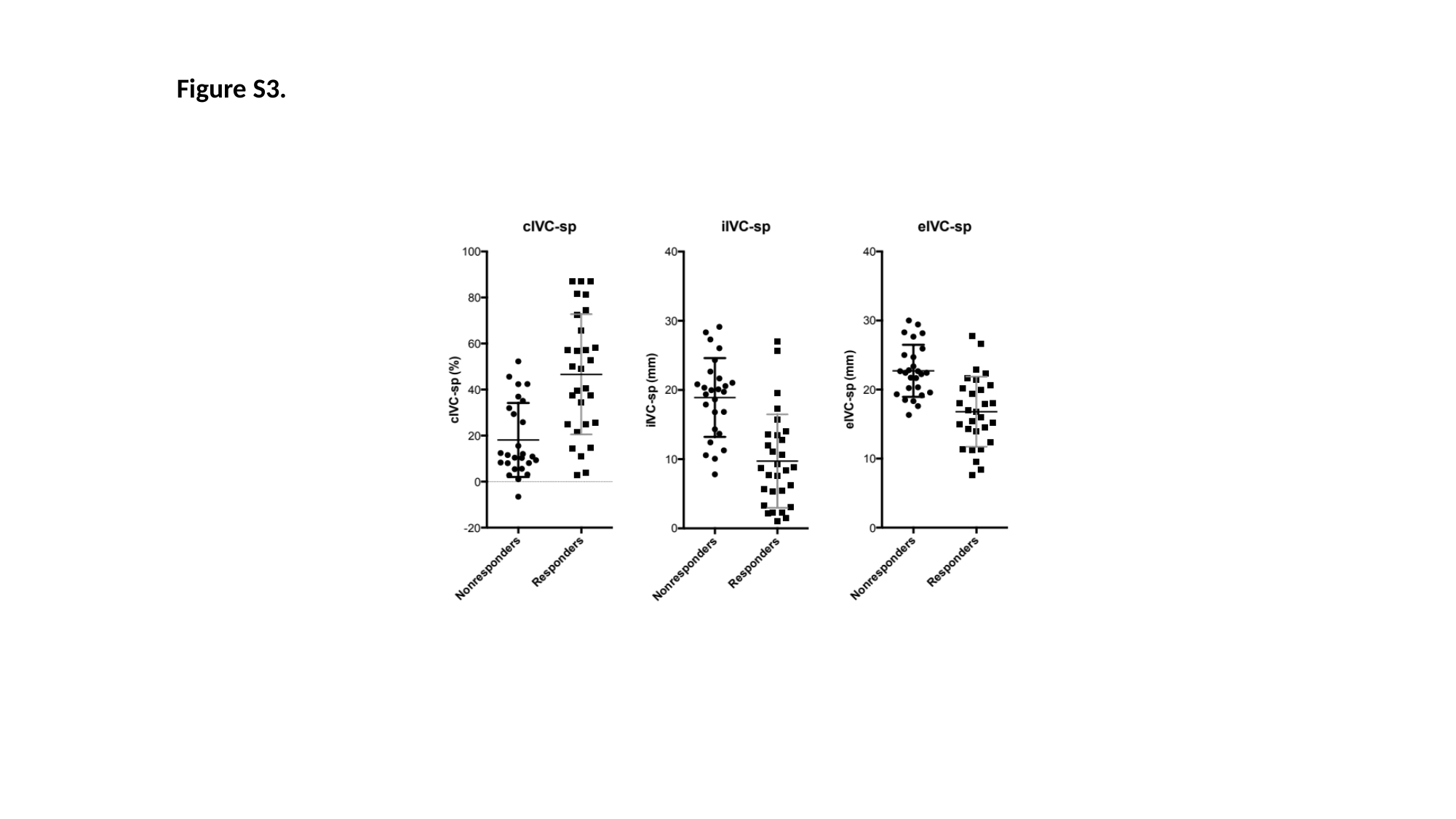

Figure S3.
