# Supplementary material for: Respiratory changes of the inferior vena cava diameter predict fluid responsiveness in spontaneously breathing patients with cardiac arrhythmias
Source: Ann Intensive Care. 2018 Aug 2;8:79. doi: 10.1186/s13613-018-0427-1 (PMC6072642; doi:10.1186/s13613-018-0427-1)
Supplement: Supplementary file 2 — Additional file 2: Table S1. Respiratory variables in responders and nonresponders before and after volume expansion. Table S2. Volume expansion-induced changes in hemodynamic variables in responders and nonresponders. Table S3. Baseline characteristics of the patients (VE-related change in VTIao ≥ 15% to define responders). Table S4. Hemodynamic variables before and after volume expansion in responders and nonresponders (VE-related change in VTIao ≥ 15% to define responders). Table S5. Accuracy of the inferior vena cava variables for predicting response to volume expansion (VE-related change in VTIao ≥ 15% to define responders). [file 13613_2018_427_MOESM2_ESM.docx]

**Additional file 2**

**Table S1. Respiratory Variables in Responders and Nonresponders before and after Volume Expansion.**

|  | Nonresponders  (n = 26) | | Responders  (n = 29) | *p* value |
| --- | --- | --- | --- | --- |
|  |  | |  |  |
| STANDARDIZED BREATHING |  | |  |  |
|  |  | |  |  |
| Minimum Pinsp, mm H_2_O | |  |  |  |
| Before VE | | -7.0 (-9.0; -5.0) | -5.0 (-8.0; -3.5) | 0.40 |
| After VE | | -7.0 (-8.3; -5.0) | -5.7 (-8.2; -4.0) | 0.43 |
| Maximum Pexp, mm H_2_O | |  |  |  |
| Before VE | | 1.0 (0.6; 2.0) | 1.0 (0.4; 2.0) | 0.44 |
| After VE | | 1.0 (0.5; 2.0) | 1.0 (0.4; 2.4) | 0.89 |
| DiaphExc, mm | |  |  |  |
| Before VE | | 15 (12; 25) | 21 (10; 26) | 0.89 |
| After VE | | 18 (12; 25) | 20 (14; 26) | 0.55 |
|  | |  |  |  |
| SPONTANEOUS VENTILATION | |  |  |  |
|  | |  |  |  |
| Minimum Pinsp, mm H_2_O | |  |  |  |
| Before VE | | -0.7 (-3.0; -0.4) | -1.0 (-2.0; -0.3) | 0.89 |
| After VE | | -1.0 (-2.0; -0.2) | -1.0 (-2.0; -0.4) | 0.83 |
| Maximum Pexp pressure, mm H_2_O | |  |  |  |
| Before VE | | 0.5 (0.2; 1.0) | 0.5 (0.2; 1.0) | 0.96 |
| After VE | | 0.4 (0.1; 0.9) | 0.6 (0.2; 1.0) | 0.27 |
| DiaphExc, mm | |  |  |  |
| Before VE | | 8 (7; 16) | 8 (5; 10) | 0.47 |
| After VE | | 8 (5; 14) | 8 (4; 13) | 0.73 |
| Respiratory rate, /min | |  |  |  |
| Before VE | | 23 ± 7 | 25 ± 6 | 0.19 |
| After VE | | 24 ± 8 | 24 ± 5 | 0.97 |

Values are expressed as mean ± standard deviation or median and interquartile range (25^th^; 75th percentiles). DiaphExc = diaphragmatic inspiratory excursion; Pexp = expiratory buccal pressure; Pinsp = inspiratory buccal pressur; VE = volume expansion. Every single value is the mean of 3 separate measurements.

**Table S2. Volume-expansion Induced Changes in Hemodynamic Variables in Responders and Nonresponders.**

|  | Nonresponders  (n = 26) | Responders  (n = 29) | *p* value  a/b |
| --- | --- | --- | --- |
| Heart rate, beats/min | -4 ± 9 | -7 ± 12 | 0.41/0.66 |
| VTIao (cm) | 0.1 ± 0.9 | 4.1 ± 2.1 | <0.0001/<0.0001 |
| SVI, mL/m² | 0 ± 2 | 8 ± 4 | <0.0001/<0.0001 |
| Systolic arterial pressure, mm Hg | 2 ± 16 | 13 ± 19 | 0.026/0.029 |
| Pulse pressure, mm Hg | 1 ± 15 | 8 ± 19 | 0.12/0.26 |
| cIVC-st, % | -8 ± 13 | -26 ± 22 | 0.001/0.66 |
| iIVC-st, mm | 3 ± 3 | 6 ± 5 | 0.013/0.70 |
| eIVC-st, mm | 2 ± 2 | 3 ± 3 | 0.42/0.56 |
|  |  |  |  |
| cIVC-sp, % | -9 ± 12 | -16 ± 21 | 0.13/0.19 |
| iIVC-sp, mm | 3 ± 4 | 5 ± 5 | 0.24/0.15 |
| eIVC-sp, mm | 2 ± 2 | 3 ± 3 | 0.044/0.71 |

Values given as mean of change ± standard deviation. IVC = inferior vena cava; cIVC-st = collapsibility index of the IVC under standardized breathing; iIVC-st = minimum-inspiratory diameter of the IVC under standardized breathing; eIVC-st = end-expiratory diameter of the IVC under standardized breathing; cIVC-sp = collapsibility index of the IVC under spontaneous breathing; iIVC-sp = minimum-inspiratory diameter of the IVC under spontaneous breathing; eIVC-sp = end-expiratory diameter of the IVC under spontaneous breathing; VTIao = velocity time integral of aortic blood flow; SVI = stroke volume index. a = unadjusted *p*-values; b = *p*-values adjusted for baseline hemodynamic variables. Every single value is the mean of 3 separate measurements.

| **Table S3. Baseline Characteristics of the Patients (VE-related change in VTIao** ≥**15% to define responders).** | | | |
| --- | --- | --- | --- |
|  | **Nonresponders**  **(n = 28)** | **Responders**  **(n = 27)** | ***p* value** |
|  |  |  |  |
| **Clinical Data** |  |  |  |
| Age, year | 70 ± 12 | 67 ± 11 | 0.35 |
| Sex ratio, female | 12 (43) | 8 (30) | 0.40 |
| Height, cm | 168 ± 9 | 171 ± 10 | 0.27 |
| Weight, kg | 77 ± 23 | 78 ± 21 | 0.96 |
| Body Mass Index, kg/m² | 27 (23; 29) | 25 (22; 29) | 0.50 |
| Admission-to-VE time (hours) | 29 (18; 41) | 18 (8; 39) | 0.31 |
|  |  |  |  |
| **Medical history** |  |  |  |
| Chronic systemic hypertension | 13 (46) | 15 (55) | 0.59 |
| Chronic left ventricular failure | 6 (21) | 8 (30) | 0.55 |
| Chronic right ventricular failure | 3 (11) | 2 (7) | 1.00 |
| Chronic obstructive pulmonary disease | 7 (25) | 7 (26) | 1.00 |
| Chronic pulmonary hypertension | 6 (21) | 2 (7) | 0.25 |
| Pulmonary embolism | 2 (7) | 1 (4) | - |
|  |  |  |  |
| **Infection** |  |  |  |
| Pulmonary infections | 15 (54) | 17 (63) | 0.59 |
| Urinary infections | 4 (14) | 1 (4) |  |
| Abdominal infections | 4 (14) | 1 (4) |  |
| Skin and soft tissue infections | 3 (11) | 5 (19) |  |
| Catheter and other infections | 2 (7) | 3 (11) |  |
|  |  |  |  |
| **Treatment** |  |  |  |
| Simplified Acute Physiology Score II | 39 ± 12 | 33 ± 12 | 0.09 |
| Norepinephrine | 7 (25) | 1 (4) | 0.051 |
| VE 24 hr before inclusion, mL | 500 (0; 1750) | 1000 (500; 1500) | 0.34 |
|  |  |  |  |
| **Clinical hemodynamic variables** |  |  |  |
| Atrial fibrillation | 16 (57) | 13 (48) | 0.59 |
| Arterial hypotension | 15 (54) | 13 (48) | 0.69 |
| Tachycardia | 20 (71) | 21 (78) | 0.81 |
| Oliguria | 12 (43) | 14 (52) | 0.50 |
| Mottled skin | 4 (14) | 8 (30) | 0.17 |
|  |  |  |  |

Values are expressed as count (percentage), mean ± standard deviation or median (25^th^; 75th percentiles). VE = volume expansion; VTIao = velocity time integral of aortic blood flow.

**Table S4. Hemodynamic Variables before and after Volume Expansion in Responders and Nonresponders (VE-related change in VTIao ≥15% to define responders).**

|  | Nonresponders  (n = 28) | Responders  (n = 27) | *p* value |
| --- | --- | --- | --- |
|  |  |  |  |
| VTIao, cm |  |  |  |
| Before VE | 15.8 ± 4.3 | 13 ± 3.6 | 0.01 |
| After VE | 16 ± 4.4 | 17.3 ± 5.1 | 0.33 |
| SVI, ml/m² |  |  |  |
| Before VE | 29 ± 8 | 24 ± 8 | 0.03 |
| After VE | 29 ± 8 | 33 ± 11 | 0.22 |
| Systolic arterial pressure, mmHg |  |  |  |
| Before VE | 110 ± 21 | 104 ± 25 | 0.34 |
| After VE | 112 ± 19 | 118 ± 23 | 0.32 |
| Pulse pressure, mmHg |  |  |  |
| Before VE | 49 ± 12 | 44 ± 18 | 0.19 |
| After VE | 50 ± 12 | 53 ± 20 | 0.57 |
| Heart rate, beats/min |  |  |  |
| Before VE | 108 ± 29 | 114 ± 25 | 0.49 |
| After VE | 104 ± 25 | 107 ± 23 | 0.61 |
|  |  |  |  |
| cIVC-st, % |  |  |  |
| Before VE | 19 (7; 34) | 77 (56; 92) | <0.0001 |
| After VE | 11 (8; 21) | 39 (24; 63) | <0.0001 |
| iIVC-st, mm |  |  |  |
| Before VE | 18 (12; 21) | 4 (1; 8) | <0.0001 |
| After VE | 20 (15; 23) | 13 (7; 16) | <0.0001 |
| eIVC-st, mm |  |  |  |
| Before VE | 22 ± 4 | 17 ± 5 | 0.002 |
| After VE | 24 ± 5 | 20 ± 5 | 0.01 |
|  |  |  |  |
| cIVC-sp, % |  |  |  |
| Before VE | 12 (8; 34) | 50 (25; 72) | <0.0001 |
| After VE | 5 (3; 15) | 25 (14; 49) | <0.0001 |
| iIVC-sp, mm |  |  |  |
| Before VE | 20 (13; 22) | 9 (3; 13) | <0.0001 |
| After VE | 21 (18; 24) | 16 (10; 18) | <0.0001 |
| eIVC-sp, mm |  |  |  |
| Before VE | 22 ± 4 | 17 ± 5 | 0.0002 |
| After VE | 24 ± 4 | 20 ± 5 | 0.008 |
|  |  |  |  |

Values are expressed as mean ± standard deviation or median (25^th^; 75th percentiles). IVC = inferior vena cava; cIVC-st = collapsibility index of the IVC under standardized breathing; iIVC-st = minimum-inspiratory diameter of the IVC under standardized breathing; eIVC-st = end-expiratory diameter of the IVC under standardized breathing; cIVC-sp = collapsibility index of the IVC under unstandardized spontaneous breathing; iIVC-sp = minimum-inspiratory diameter of the IVC under unstandardized spontaneous breathing; eIVC-sp = end-expiratory diameter of the IVC under unstandardized spontaneous breathing; SVI = stroke volume index; VE= volume expansion; VTIao= velocity time integral of aortic blood flow. Every single value is the mean of 3 separate measurements.

**Table S5. Accuracy of the Inferior Vena Cava Variables for Predicting Response to Volume Expansion (VE-related change in VTIao ≥15% to define responders).**

| **Variables** | **Area Under**  **ROC Curve**  [95% CI] | | | **Threshold** | | **Sensitivity**  [95% CI] | | **Specificity**  [95% CI] | | **Positive predictive value**  (%) | | **Negative predictive value**  (%) |
| --- | --- | --- | --- | --- | --- | --- | --- | --- | --- | --- | --- | --- |
|  |  | | |  | |  | |  | |  | |  |
| cIVC-st | 0.93 [0.86; 1.00] | | | **≥51*** | | 0.85 [0.66; 0.96] | | 0.96 [0.81; 1] | | 96 | | 87 |
| % |  | | | ≥40 | | ≥0.9 | |  | | 83 | | 89 |
|  |  | | | ≥48 | |  | | ≥0.9 | | 92 | | 86 |
| iIVC-st | 0.91 [0.84; 0.99] | | | **<9*** | | 0.78 [0.58; 0.91] | | 0.93 [0.76; 0.99] | | 92 | | 81 |
| mm |  | | | <13 | | ≥0.9 | |  | | 73 | | 86 |
|  |  | | | <9 | |  | | ≥0.9 | | 88 | | 80 |
| eIVC-st | 0.72 [0.59; 0.86] | | | **<20*** | | 0.65 [0.46; 0.84] | | 0.70 [0.50; 0.86] | | 68 | | 67 |
| mm |  | | | <24 | | ≥0.9 | |  | | 57 | | 80 |
|  |  | | | <16 | |  | | ≥0.9 | | 80 | | 60 |
|  |  | | |  | |  | |  | |  | |  |
| cIVC-sp | 0.81 [0.70; 0.93] | | | **≥37*** | | 0.67 [0.46; 0.84] | | 0.82 [0.63; 0.94] | | 79 | | 71 |
| % |  | | | ≥11 | | ≥0.9 | |  | | 63 | | 86 |
|  |  | | | ≥43 | |  | | ≥0.9 | | 86 | | 67 |
| iIVC-sp | 0.84 [0.73; 0.95] | | | **<11*** | | 0.67 [0.46; 0.84] | | 0.86 [0.67; 0.96] | | 83 | | 72 |
| mm |  | | | <20 | | ≥0.9 | |  | | 65 | | 87 |
|  |  | | | <10 | |  | | ≥0.9 | | 86 | | 69 |
| eIVC-sp | 0.78 [0.66; 0.90] | | | **<18*** | | 0.63 [0.43; 0.81] | | 0.88 [0.72; 0.98] | | 84 | | 70 |
| mm |  | | | <23 | | ≥0.9 | |  | | 61 | | 79 |
|  |  | | | <16 | |  | | ≥0.9 | | 83 | | 61 |
|  | |  |  | |  | |  | |  | |  | |

CI = confidence interval; IVC = inferior vena cava; cIVC-st = collapsibility index of the IVC under standardized breathing; iIVC-st = minimum-inspiratory diameter of the IVC under standardized breathing; eIVC-st = end-expiratory diameter of the IVC under standardized breathing; cIVC-sp = collapsibility index of the IVC under unstandardized spontaneous breathing; iIVC-sp = minimum-inspiratory diameter of the IVC under unstandardized spontaneous breathing; eIVC-sp = end-expiratory diameter of the IVC under unstandardized spontaneous breathing; ROC = Receiver Operating Characteristic; VE = volume expansion; VTIao = velocity time of aortic blood flow. * optimal threshold value to predict response to VE.
